# Epidemiology and Survival of Patients With Optic Pathway Gliomas: A Population-Based Analysis

**DOI:** 10.3389/fonc.2022.789856

**Published:** 2022-02-11

**Authors:** Huanbing Liu, Yong Chen, Xiaowei Qin, Zheng Jin, Yining Jiang, Yubo Wang

**Affiliations:** Department of Neurosurgery, First Affiliated Hospital of Jilin University, Changchun, China

**Keywords:** epidemiology, survival, SEER program, CNS disease, optic pathway glioma

## Abstract

**Background:**

We aimed to analyze the epidemiology and outcomes of pediatric patients and adult patients with optic pathway gliomas in the United States using a population-based method.

**Methods:**

Data for patients with optic pathway gliomas diagnosed between 2000 and 2018 were extracted from the SEER database. We divided the patients into a pediatric group and an adult group. Descriptive analyses were conducted to analyze demographic and clinical characteristics and treatment. We used the chi-square test to evaluate differences between pediatric and adult patients with optic pathway gliomas. The possible prognostic indicators were analyzed by Kaplan–Meier curves and Cox proportional hazards models.

**Results:**

Optic pathway gliomas represented 86.6% of all lesions originating from the optic pathway. In total, 1257 cases of optic pathway gliomas were included in our study. Pediatric patients accounted for 83.7% in this cohort, and most of the patients were diagnosed at 1-4 years old. Chemotherapy was chosen most often for pediatric patients, but radiation therapy was chosen most often for adult patients. Pilocytic astrocytoma accounted for 59.1% of pediatric patients and 37.5% of adult patients. The overall survival (OS) rates were 94.8% 5 years after diagnosis and 93.0% 10 years after diagnosis. Survival analysis showed that surgery, radiation and chemotherapy did not help patients obtain a better prognosis. Overall, pediatric patients had a better prognosis.

**Conclusion:**

Optic pathway gliomas are relatively rare lesions with good prognosis. They mostly affect children, and pilocytic astrocytoma is the most common histological diagnosis. Highly individualized treatment is essential for such patients.

## Introduction

Gliomas account for almost 30% of all primary brain tumors and are responsible for the majority of deaths from primary brain tumors (1). Optic pathway gliomas (OPGs), also known as optic nerve gliomas, are relatively rare lesions that comprise 1% of all intracranial tumors and 3–5% of all pediatric brain tumors (2, 3). OPGs are believed to be the most common tumor of the optic nerve, and they are confined to the structures of the visual pathway (2, 4, 5). The Surveillance, Epidemiology, and End Results (SEER) Program is a clinical database funded by the National Cancer Institute (NCI) that was created to collect cancer incidence, prevalence, and survival data in the United States, covering approximately 35% of the United States population (6). We conducted this population-based study to analyze the epidemiology and outcome of patients with OPGs using data from the SEER program.

## Method

### Detailed Clinical Data Extraction

The SEER database is available to the public for research purposes, and no ethics committee approval or informed consent was required to perform this analysis. Patients diagnosed with glioma originating from the optic pathway (C73.0-optic nerve) from 2000 to 2018 were included. The term glioma was defined by setting the variable “Histology recode - broad groupings” as “9380-9489: gliomas”. SEER*Stat (Surveillance Research Program, National Cancer Institute SEER*Stat software version 8.3.9) was used to extract detailed patient data from SEER Research Plus Data, 18 Registries (November 2020 submission) (7).

### Variables and Population Analysis

Demographic and clinical variables included age at diagnosis (0-19 years and older than 19 years), sex (male, female), race (white, other), laterality (left, right, bilateral and unknown), behavior code (benign and borderline, malignant), surgery (yes, none/unknown), radiation therapy (yes, none/unknown), chemotherapy (yes, none/unknown), survival months and vital status (alive, dead). First, we analyzed the distribution of patients by age at diagnosis. Second, we divided the patients into pediatric and adult groups and evaluated the differences in demographic and clinical characteristics and treatment between pediatric and adult patients with OPGs by the chi-square test, and statistical significance was set to p<0.05. Third, we analyzed the differences in treatment patterns between the two groups. Fourth, we analyzed the distribution of patients by pathology type, and pathology type was recorded according to the code “Histology recode - Brain groupings”. Only patients with available histology were included to make the results more accurate.

### Survival Analysis

Survival to 5 and 10 years after diagnosis was calculated using the Kaplan–Meier method. The log-rank test and univariate Cox proportional hazard models were performed to estimate possible independent prognostic factors associated with overall survival (OS) in patients with OPGs, and statistical significance was set to p<0.05. OS was defined as the time from diagnosis to death from any cause. All the data were analyzed using SPSS Statistics, Version 25 (IBM SPSS Statistics for Windows, Version 25 Armonk, NY: IBM Corp). Survival analysis was first conducted for the entire cohort. To make the sample more homogeneous, survival analysis was performed for pediatric patients and adult patients separately.

## Results

### Population Analysis

**Figure 1:**
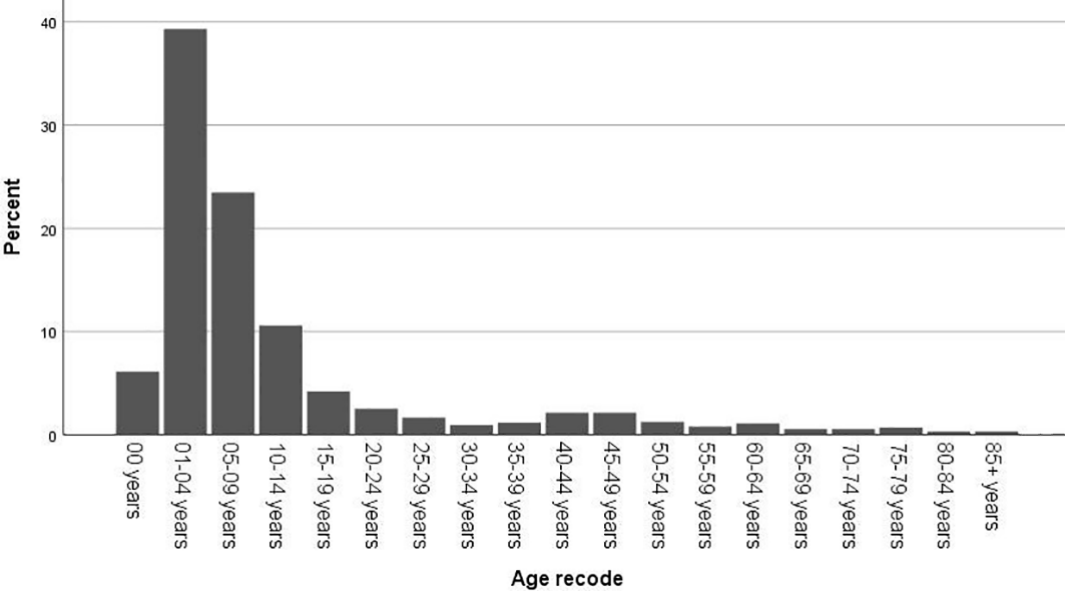
Distribution of the patients by age at diagnosis.

In total, 1451 cases of primary lesions originating from the optic nerve were indexed between 2000 and 2018, and 1257 cases were identified as gliomas, which represented 86.6% of all patients with lesions originating from the optic nerve. Pediatric patients (≤19 years old) accounted for 83.7% of the cohort, and most of the patients were diagnosed at 1-4 years old (**Figure 1**). There were 662 female patients (52.7%) and 595 male patients (47.3%), and white patients accounted for approximately 82.8% (n=1041) of all patients. Overall, 43.4% of the tumors (n=89) originated from the left side in adult patients, and 41.6% of the tumors (n=438) were bilateral or unknown in pediatric patients. There was a statistically significant difference between the pediatric and adult populations in laterality and treatment options (**Table 1**). A total of 63.1% of the patients (n=793) chose observation for treatment. Except for observation, pediatric patients mostly chose chemotherapy, and adult patients mostly chose radiation therapy (**Table 2**). We analyzed the pathology type of the patients with available histology, and 283 cases were included (80 adults and 203 pediatric patients). Pilocytic astrocytoma accounted for 53.0% of the patients with available histology (59.1% for pediatric patients and 37.5% for adult patients). Overall, 17.5% of the adult patients were diagnosed with glioblastoma, and no pediatric patients were diagnosed with glioblastoma (**Table 3**).

**Table 1:** Demographic and clinical characteristics of patients with optic pathway gliomas.

Variables	Adult	Pediatric	Total	P value
**Sex**				0.091
Female	119(58.0%)	543(51.6%)	662(52.7%)	
Male	86(42.0%)	509(48.4%)	595(47.3%)	
**Race**				0.334
White	165(80.5%)	876(83.3%)	1041(82.8%)	
Others	40(19.5%)	176(16.7%)	216(17.2%)	
**Laterality**				<0.001
Right	89(43.4%)	313(29.8%)	402(32.0%)	
Left	63(30.7%)	301(28.6%)	364(29.0%)	
Biliteral and unknown	53(25.9%)	438(41.6%)	491(39.1%)	
**Behavior code**				0.461
Benign and borderline	14(6.8%)	88(8.4%)	102(8.1%)	
Malignant	191(93.2%)	964(91.6%)	1155(91.9%)	
**Surgery**				<0.001
Yes	40(19.5%)	111(10.6%)	151(12.0%)	
None/Unknown	165(80.5%)	941(89.4%)	1106(88.0%)	
**Radiation**				<0.001
Yes	76(37.1%)	19(1.8%)	95(7.6%)	
None/Unknown	129(62.9%)	1033(98.2%)	1162(92.4%)	
**Chemotherapy**				<0.001
Yes	30(14.6%)	298(28.3%)	328(26.1%)	
None/Unknown	175(85.4%)	754(71.7%)	929(73.9%)	

**Table 2:** Analysis of treatment patterns chosen by patients with optic pathway gliomas.

Treatment	Adult	Pediatric	Total
Chemotherapy	8(3.9)	237(22.5)	245(19.5)
Chemotherapy and Radiation	13(6.3)	4(0.4)	17(1.4)
Observation	101(49.3)	692(65.8)	793(63.1)
Radiation	43(21.0)	8(0.8)	51(4.1)
Surgery	19(9.3)	51(4.8)	70(5.6)
Surgery and Chemotherapy	1(0.5)	53(5.0)	54(4.3)
Surgery and Radiation	12(5.9)	3(0.3)	15(1.2)
Surgery+Radiation+Chemotherapy	8(3.9)	4(0.4)	12(1.0)
Total	205(100.0)	1052(100.0)	1257(100.0)

**Table 3:** The distribution of the patients with histology records.

Histology recode	Adult	Pediatric	Total
Anaplastic astrocytoma	7(8.8)	1(0.5)	8(2.8)
Astrocytoma, NOS	10(12.5)	13(6.4)	23(8.1)
Benign and malignant neuronal/glial, neuronal and mixed	0	1(0.5)	1(0.4)
Diffuse astrocytoma (protoplasma, fibrillary)	0	1(0.5)	1(0.4)
Embryonal/primitive/medulloblastoma	0	1(0.5)	1(0.4)
Ependymoma/anaplastic ependymoma	1(1.3)	0	1(0.4)
Glioblastoma	14(17.5)	0	14(4.9)
Glioma, NOS	18(22.5)	64(31.5)	82(29.0)
Mixed glioma	0	2(1.0)	2(0.7)
Pilocytic astrocytoma	30(37.5)	120(59.1)	150(53.0)
Total	80(100.0)	203(100.0)	283(100.0)

### Survival Analysis

**Figure 2:**
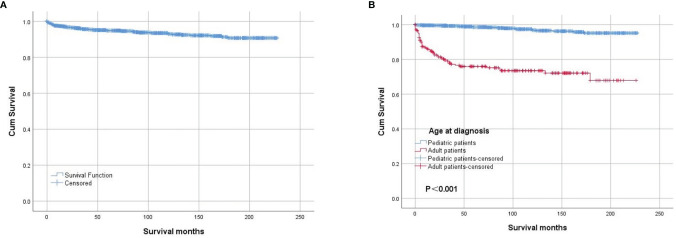
Kaplan–Meier survival analysis: **(A)** The overall survival for the whole cohort. The survival analysis of patients classified based on age at diagnosis **(B)**.

The OS rates were 94.8% 5 years after diagnosis and 93.0% 10 years after diagnosis. The OS of the whole cohort is shown in **Figure 2A**, as determined by a Kaplan–Meier curve. The results of the log-rank test indicated that pediatric patients had better OS than adult patients (**Figure 2B**). The results for the log-rank tests and univariate Cox proportional hazard models for the whole cohort showed that sex, race, laterality and behavior code did not statistically significantly influence OS. Meanwhile, we found that treatments, including surgery, radiation and chemotherapy, did not result in better prognoses. Then, we analyzed the pediatric patients and adult patients separately to avoid heterogeneity, but the results were unchanged. The results of the log-rank test are presented in **Table 4**, and the results of the univariate Cox proportional hazard models, including the hazard ratios (HRs) and 95% confidence intervals (CIs), are presented in **Table 5**.

**Table 4:** The results of the log-rank test.

Variables	Adult	Pediatric	Total
**Age at diagnosis** (years)			<0.001
0-19			
>19			
**Sex**	0.191	0.814	0.814
Female			
Male			
**Race**	0.931	0.461	0.517
White			
Others			
**Laterality**	0.654	0.688	0.779
Right			
Left			
Biliteral and unknown			
**Behavior code**	0.931	0.903	0.375
Benign and borderline			
Malignant			
**Surgery**	0.081	<0.001	<0.001
Yes			
None/Unknown			
**Radiation**	<0.001	<0.001	<0.001
Yes			
None/Unknown			
**Chemotherapy**	<0.001	0.068	0.013
Yes			
None/Unknown			

**Table 5:** The results of the univariate Cox regression analysis.

Variables	Adult		Pediatric		Total	
	HR(95%CI)	P value	HR(95%CI)	P value	HR(95%CI)	P value
**Age at diagnosis** (years)						
0-19					0.082(0.050-0.134)	<0.001
>19					Reference	
**Sex**						
Female	Reference		Reference		Reference	
Male	1.443(0.829-2.513)	0.195	0.908(0.407-2.028)	0.814	1.056(0.669-1.666)	0.815
**Race**						
White	Reference		Reference		Reference	
Others	1.032(0.501-2.125)	0.932	1.445(0.539-3.874)	0.464	1.212(0.677-2.169)	0.518
**Laterality**						
Right	0.935(0.461-1.896)	0.853	0.709(0.253-1.988)	0.513	1.147(0.651-2.021)	0.635
Left	0.743(0.376-1.468)	0.393	0.685(0.245-1.920)	0.472	1.208(0.700-2.087)	0.497
Biliteral and unknown	Reference		Reference		Reference	
**Behavior code**						
Benign and borderline	Reference		Reference		Reference	
Malignant	3.162(0.436-22.915)	0.255	0.882(0.118-6.608)	0.903	1.870(0.458-7.644)	0.383
**Surgery**						
Yes	1.704(0.927-3.133)	0.086	5.515(2.463-12.348)	<0.001	3.495(2.165-5.642)	<0.001
None/Unknown	Reference		Reference		Reference	
**Radiation**						
Yes	2.743(1.555-4.836)	<0.001	7.142(2.430-20.985)	<0.001	11.219(7.099-17.729)	<0.001
None/Unknown	Reference		Reference		Reference	
**Chemotherapy**						
Yes	5.436(3.010-9.816)	<0.001	2.075(0.931-4.627)	0.074	1.788(1.123-2.845)	0.014
None/Unknown	Reference		Reference		Reference	

## Discussion

OPGs are relatively rare and mostly affect children (8). Several systematic reviews have been reported (5, 9), but population-based studies of OPGs have seldom been described. The SEER database provides a sufficient amount of publicly available information for research purposes, enabling us to conduct this population-based analysis to better understand OPGs. To ensure that sufficient information was included, we chose the latest database and set the period as 2000-2018.

In our study, we found that more than 80% of the patients were diagnosed before 20 years of age. However, the oldest patient was 101 years old, and OPGs can be diagnosed at any age. Survival analysis showed that age at diagnosis was an independent prognostic factor, and pediatric patients had a better prognosis than adult patients. The predominance of white patients was also noted, as in other reports (10). It has been reported that 85% of OPGs are located in the optic nerves and/or chiasm, and 15% are located in the optic tracts and radiation (11). In addition, chiasmatic/hypothalamic tumor sites have been reported as risk factors for long-term visual deterioration (12). We could not obtain information on whether the OPGs originated from the optic nerve, chiasm or optic tracts. Therefore, we analyzed laterality instead, and we found that 41.6% of the tumors in the pediatric group did not originate from the left or right. We deduced that these tumors originated from the chiasm. In addition, the influence of laterality was not significant in the survival analysis; further high-quality studies are needed to address the potential role of laterality at the time of OPG presentation on OS.

Although most of these tumors were pilocytic astrocytomas, we also found that some of the OPGs were glioblastomas, accounting for 17.5% of the adult patients with available histology. Because only 22.5% of the diagnoses were confirmed by available histology, we did not include pathology type in the survival analysis. In 2020, Kinori et al. (13) reported that children with neurofibromatosis type 1 (NF-1)-associated OPGs who had a normal initial exam had excellent long-term visual function. Pediatric patients with sporadic OPGs, however, have been reported to have significant long-term visual impairment (14). A systematic review from Opocher et al. (5) showed that solid evidence is needed to prove whether NF-1 is an independent prognostic factor. Unfortunately, information on NF-1 was not included in the SEER program; thus, we could not conduct such an analysis. However, our survival analysis results showed that pediatric patients have better OS than their adult counterparts.

The treatment for OPGs remains controversial (9, 15, 16). According to a previous report, observation is suggested for patients with neurofibromatosis type 1 (NF-1) or nonprogressive gliomas. When pronounced proptosis and blindness are present or a mass effect or hydrocephalus is observed, surgery is usually considered appropriate (15, 16). Our results show that most (63.1%) of the treatment regimens were no/unknown because the codes for radiation and chemotherapy in the SEER database did not distinguish between “no” and “unknown” treatment. However, the surgery codes showed that only 14 of the 1257 cases (1.1%) were unknown. Therefore, we speculate that observation is recommended for most patients. The survival analysis revealed that the patients who underwent surgery had a worse prognosis. We speculate that the patients had larger tumors or more serious visual damage; thus, although surgery was conducted, the prognosis was not better than that of their counterparts.

Because radiation-related complications, including endocrinopathy, vasculopathy, and cognitive decline, occur in young children, radiation therapy has been gradually abandoned for pediatric patients with OPGs, and chemotherapy has been increasingly adopted (17). Our result of the treatment analysis was coincident with previous reports, but the patients who received chemotherapy also had a worse prognosis. Moreno et al. (9) conducted a systematic review in 2010 and found that treatment with chemotherapy does not improve the resulting vision in the majority of children with OPGs. However, indication bias may exist in our study. Perhaps the worse survival of patients with treatment is due to the severity of the tumor itself and not the treatment. We agree that the treatment of OPGs requires a multidisciplinary approach in which all treatment options are implemented in a highly individualized manner (18).

Except for possible bias and inaccurate data, we have to consider other limitations of our analysis. First, information about tumor progression and ophthalmologic examinations, such as visual acuity and visual field, was limited and is very important for patients with OPGs. Second, the SEER program provided limited information about the genetics of central nervous system tumors, and NF-1 is believed to be associated with the survival of patients with OPGs (19). Third, detailed information about surgery, radiation and chemotherapy was limited, and our results of the survival analysis therefore could not explain the specific condition of individual patients. Despite these limitations, this population-based study can provide helpful information and a better understanding of the epidemiology and survival of patients with OPGs.

## Conclusion

OPGs are relatively rare lesions, representing 86.6% of all patients with lesions originating from the optic pathway. They mostly affect children, and pilocytic astrocytoma is the most common histological diagnosis. In our cohort, the prognosis was good, and the OS rate at 10 years after diagnosis was 93.0%. Observation is recommended for most patients. Based on the SEER data, surgery, radiation and chemotherapy showed no evidence of improving OS. Highly individualized treatment is essential for patients with OPGs.

## Data Availability Statement

The raw data supporting the conclusions of this article will be made available by the authors, without undue reservation.

## Ethics Statement

Ethical review and approval was not required for the study on human participants in accordance with the local legislation and institutional requirements. Written informed consent from the participants’ legal guardian/next of kin was not required to participate in this study in accordance with the national legislation and the institutional requirements.

## Author Contributions

YW: Conceptualization, methodology, and writing—reviewing and editing. ZJ, YJ, and YW: Data curation, software, and validation. HL, YC, XQ, and YW: Writing—original draft preparation. Writing—reviewing and editing. All authors contributed to the article and approved the submitted version.

## Conflict of Interest

The authors declare that the research was conducted in the absence of any commercial or financial relationships that could be construed as a potential conflict of interest.

## Publisher’s Note

All claims expressed in this article are solely those of the authors and do not necessarily represent those of their affiliated organizations, or those of the publisher, the editors and the reviewers. Any product that may be evaluated in this article, or claim that may be made by its manufacturer, is not guaranteed or endorsed by the publisher.
